# Quadruplet-Based Deep Cross-Modal Hashing

**DOI:** 10.1155/2021/9968716

**Published:** 2021-07-02

**Authors:** Huan Liu, Jiang Xiong, Nian Zhang, Fuming Liu, Xitao Zou

**Affiliations:** ^1^Key Laboratory of Intelligent Information Processing and Control, Chongqing Municipal Institutions of Higher Education, Chongqing Three Gorges University, Chongqing 40044, China; ^2^Department of Electrical and Computer Engineering, University of the District of Columbia, Washington, D. C., SC 20008, USA

## Abstract

Recently, benefitting from the storage and retrieval efficiency of hashing and the powerful discriminative feature extraction capability of deep neural networks, deep cross-modal hashing retrieval has drawn more and more attention. To preserve the semantic similarities of cross-modal instances during the hash mapping procedure, most existing deep cross-modal hashing methods usually learn deep hashing networks with a pairwise loss or a triplet loss. However, these methods may not fully explore the similarity relation across modalities. To solve this problem, in this paper, we introduce a quadruplet loss into deep cross-modal hashing and propose a quadruplet-based deep cross-modal hashing (termed QDCMH) method. Extensive experiments on two benchmark cross-modal retrieval datasets show that our proposed method achieves state-of-the-art performance and demonstrate the efficiency of the quadruplet loss in cross-modal hashing.

## 1. Introduction

With the advent of the era of big data, there are surging massive multimedia data on the Internet, such as images, videos, and texts. These data usually exist in diversified modalities, for example, there may exist a textual data and an audio data describing a video data or an image data. As data from different modalities may have compact semantic relevance, cross-modal retrieval [1, 2] is proposed to retrieve semantic similar data from one modality while the querying data is from a distinct modality. Benefitting from the high efficiency and low cost, hashing-based cross-modal retrieval (cross-modal hashing) [3–6] has drew extensive attention. The goal of cross-modal hashing is to map the modal heterogeneous data into a common binary space and ensure that semantic similar/dissimilar cross-modal data have similar/dissimilar hash codes. Cross-modal hashing methods can usually achieve superior performance; nonetheless, most of existing cross-modal hashing methods (such as cross-modal similarity sensitive hashing (CMSSH) [7], semantic correlation maximization (SCM) [8], semantics-preserving hashing (SePH) [9], and generalized semantic preserving hashing (GSPH) [10]) are based on handcrafted feature learning, which cannot effectively capture the heterogeneous relevance between different modalities and thus may result in inferior performance.

In the last decade, deep convolutional neural networks [11, 12] have been successfully utilized in many computer vision tasks, and therefore, some researchers also deploy it in cross-modal hashing, such as deep cross-modal hashing (DCMH) [13], pairwise relationship guided deep hashing (PRDH) [14], self-supervised adversarial hashing (SSAH) [15], and triplet-based deep hashing (TDH) [16]. Cross-modal hashing methods with deep neural networks efficiently integrate the hash representation learning and the hash function learning into an end-to-end framework, which can capture heterogeneous cross-modal relevance more effectively and thus acquire better cross-modal retrieval performance.

**Figure 1:**
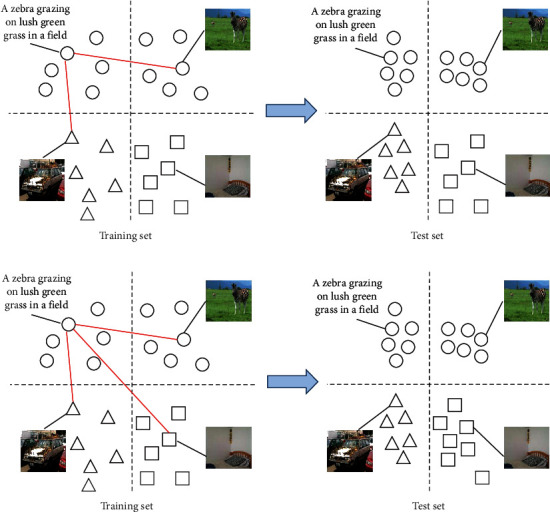
(a) Triplet loss-based cross-modal hashing methods suffer from a weak generalization capacity from the training set to the testing set because the test instances belong to the category 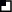 and cannot be mapped into compact binary codes (see the lower-right corner). (b) Triplet loss-based cross-modal hashing methods can project the test instances, which belong to the category 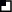, into compact binary space (see the lower right corner).

To date, most deep cross-modal hashing methods utilize the pairwise loss (such as [13–15]) or the triplet loss (such as [16]) to preserve semantic relevance during the hash representation learning procedure. Nevertheless, the pairwise loss- and triplet loss-based hash methods suffer from a weak generalization capacity from the training set to the testing set [17, 18], as shown in Figure 1(a). On the contrary, quadruplet loss is proposed and has been utilized in image hashing retrieval [17] and person reidentification [18], and in these works, it has been proved that the quadruplet loss-based model can enhance the generalization capability. Therefore, cross-modal hashing combines quadruplet loss as a natural solution to enhance the performance of cross-modal hashing, as shown in Figure 1(b).

To this end, in this paper, we introduce quadruplet loss into cross-modal hashing and propose a quadruplet-based deep cross-modal hashing method (QDCMH). Specifically, QDCMH firstly defines a quadruplet-based cross-modal semantic preserving module. Afterwards, QDCMH integrates this module, hash representation learning, and hash code generation into an end-to-end framework. Finally, experiments on two benchmark cross-modal retrieval datasets are conducted to validate the performance of the proposed method. The main contributions of our proposed QDCMH include the following:

- We introduce quadruplet loss into cross-modal retrieval and propose a novel deep cross-modal hashing method. To the best of our knowledge, this is the first work to introduce quadruplet loss into cross-modal hashing retrieval.
- We conduct extensive experiments on benchmark cross-modal retrieval datasets to investigate the performance of our proposed QDCMH.

The remainder of this paper is organized as follows.  Section 2 elaborates our proposed quadruplet-based deep cross-modal hashing method.  Section 3 presents the learning algorithm of QDCMH.  Section 4 is the experimental results and the corresponding analysis.  Section 5 concludes our work.

## 2. Proposed Method

**Figure 2:**
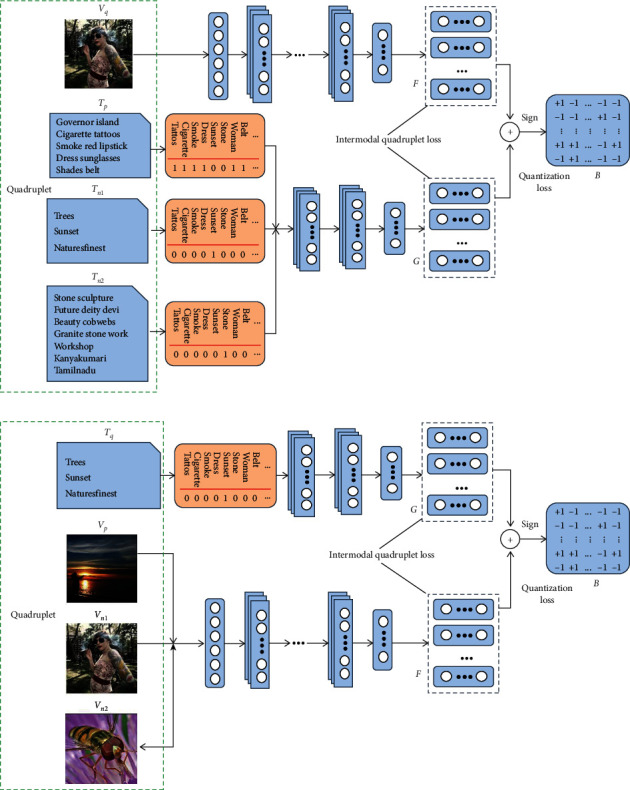
Flowchart of the proposed quadruplet-based deep cross-modal hashing (QDCMH) method. QDCMH encompasses three steps: (1) a quadruplet-based cross-modal semantic preserving module, (2) a classical convolutional neural network is used to learn image-modality features and the TxtNet in SSAH [15] is adopted to learn the text-modality features, and (3) an intermodal quadruplet loss is utilized to efficiently capture the relevant semantic information during the feature learning process and a quantization loss is used to decrease information loss during the hash codes generation procedure. (a) Quadruplet (*V*_*q*_, *T*_*p*_, *T*_*n*1_, *T*_*n*2_), which utilizes an image instance *V*_*q*_ to retrieve three text instances: *T*_*p*_, *T*_*n*1_, and *T*_*n*2_. *V*_*q*_ and *T*_*p*_ have at least one common labels, while *V*_*q*_ and *T*_*n*1_, *V*_*q*_ and *T*_*n*2_, and *T*_*n*1_ and *T*_*n*2_ are three pairwise instances and the two instances in each pairwise have no common label. (b) Quadruplet (*V*_*q*_, *T*_*p*_, *T*_*n*1_, *T*_*n*2_), which utilizes a text instance *T*_*q*_ to retrieve three image instances: *V*_*p*_, *V*_*n*1_, and *V*_*n*2_. *T*_*q*_ and *V*_*p*_ have at least one common labels, while *T*_*q*_ and *V*_*n*1_, *T*_*q*_ and *V*_*n*2_, and *V*_*n*1_ and *V*_*n*2_ are three pairwise instances and the two instances in each pairwise have no common label.

In this section, we elaborate our proposed quadruplet-based deep cross-modal hashing (QDCMH) method with the following sections: notations, quadruplet-based cross-modal semantic preserving module, feature learning networks, and hash function learning.  Figure 2 presents the flowchart of our proposed QDCMH, which cooperates quadruplet-based cross-modal semantic preserving module, hash representation learning, and hash codes generation into an end-to-end framework. In our proposed QDCMH method, we assume that each instance has two modalities, i.e., an image modality and a text modality, but they can be easily applied to multimodalities.

### 2.1. Notations

Assume that the training data comprises *n* image-text pairs, i.e., the original image features *V* ∈ *R*^*n*×*d*_*v*_^ and the original text features *T* ∈ *R*^*n*×*d*_*t*_^. Besides, there is a label vector associated with each image-text pair and label vectors for all training instances constitute a label matrix *L* ∈ *R*^*n*×*d*_*l*_^. *d*_*v*_ and *d*_*t*_ are the corresponding original dimensions of image features and text features, respectively, and *d*_*l*_ is the total number of class categories. If image-text pair {*V*_*i*_, *T*_*i*_} attaches to the *j*th category, then *L*_*ij*_=1, otherwise *L*_*ij*_=0. The quadruplet (*V*_*q*_, *T*_*p*_, *T*_*n*1_, *T*_*n*2_) denotes that *V*_*q*_ is a query instance from the image modality, and *T*_*p*_, *T*_*n*1_, *T*_*n*2_ are three retrieval instances from the text modality, where *V*_*q*_ and *T*_*p*_ have at least one common categories, while *V*_*q*_ and *T*_*n*1_, *V*_*q*_ and *T*_*n*2_, and *T*_*n*1_ and *T*_*n*2_ are three pairwise instances and the two instances in each pairwise have no common label.

With the known quadruplet (*V*_*q*_, *T*_*p*_, *T*_*n*1_, *T*_*n*2_), the target of our proposed QDCMH is to learn the corresponding hash codes (*B*_*V*_*q*__, *B*_*T*_*p*__, *B*_*T*_*n*1__, *B*_*T*_*n*2__), where *B*_*V*_*q*__, *B*_*T*_*p*__, *B*_*T*_*n*1__, *B*_*T*_*n*2__ are the hash codes of instances *V*_*q*_, *T*_*p*_, *T*_*n*1_, *T*_*n*2_, respectively. To learn the above hash codes, we first learn the hash representations (*F*_*V*_*q*__, *G*_*T*_*p*__, *G*_*T*_*n*1__, *G*_*T*_*n*2__) from the quadruplet (*V*_*q*_, *T*_*p*_, *T*_*n*1_, *T*_*n*2_) with deep neural networks, where *F*_*V*_*q*__=*f*(*V*_*q*_, *θ*_*V*_) and *G*_*T*_*p*__=*g*(*T*_*p*_, *θ*_*T*_) are the hash representations of instance *V*_*q*_ and *T*_*p*_, respectively. *f*(., *θ*_*V*_) and *g*(., *θ*_*T*_) are the hash representation learning functions for the image modality and the text modality, respectively. *θ*_*V*_ and *θ*_*T*_ are the parameters of deep neural networks to extract features for the image modality and for the text modality, respectively. Secondly, we can utilize the following sign function to approximately map the hash representations into the corresponding hash codes, i.e., *B*_*V*_*q*__=sign(*F*_*V*_*q*__) and *B*_*T*_*p*__=sign(*G*_*T*_*p*__). In the same way, we can learn the hash codes of quadruplet (*T*_*q*_, *V*_*p*_, *V*_*n*1_, *V*_*n*2_). For convenience, we denote the hash codes of all training image-text pairs, the hash representations of all training image instances, and the hash representations of all training text instances as *B* ∈ {−1,1}^*n*×*k*^, *F* ∈ *R*^*n*×*k*^, and *G* ∈ *R*^*n*×*k*^, respectively, where *k* is the length of hash codes:

(1) $$\begin{array}{c} y=\left\{\begin{array}{cc} 1, & \text{if }x>=0,x\in R,\\ -1, & \text{if }x<0,x\in R. \end{array}\right) \end{array}$$

### 2.2. Quadruplet-Based Cross-Modal Semantic Preserving Module

In cross-modal hashing retrieval, given an image instance *V*_*i*_ and a text instance *T*_*j*_, it is intractable to preserve the semantic relativity during the hash code learning procedure as the huge semantic gap across modalities. To solve this, DCMH [13] defines pairwise loss to map similar/dissimilar image-text pairs into similar/dissimilar hash codes. TDH [16] utilizes triplet loss to learn similar hash codes for similar cross-modal instances and generate distinct hash codes for semantic irrelevant cross-modal instances. Both pairwise loss and triplet loss can preserve the relevance in the original instance space; however, pairwise loss- and triplet loss-based hashing methods often suffer from a weaker generalization capability from the training set to the testing set [17, 18]. To solve this problem, in this section, a quadruplet-based cross-modal semantic preserving module is proposed to boost the generalization capability and better preserve the semantic relevance for cross-modal hashing.

For a quadruplet (*V*_*q*_, *T*_*p*_, *T*_*n*1_, *T*_*n*2_), we should keep the semantic relevance unchanged during the hash representation learning, i.e., *F*_*V*_*q*__ should be similar to *G*_*T*_*p*__, *F*_*V*_*q*__ should be distinct to *G*_*T*_*n*1__ and *G*_*T*_*n*2__, and *G*_*T*_*n*1__ should be dissimilar with *G*_*T*_*n*2__. Thus, we can define the following quadruplet loss for cross-modal hashing:

(2) $$\begin{array}{c} J_{\text{quadruplet}}^{I⟶T}\left(F_{V_{q}},G_{T_{p}},G_{T_{n1}},G_{T_{n2}}\right)=\sum_{V_{q},T_{p},T_{n1}}\max\left(0,{\left\|F_{V_{q}}-G_{T_{p}}\right\|}_{2}^{2}-{\left\|F_{V_{q}}-G_{T_{n1}}\right\|}_{2}^{2}+\alpha_{1}\right)\\ +\sum_{V_{q},T_{p},T_{n1},T_{n2}}\max\left(0,{\left\|F_{V_{q}}-G_{T_{p}}\right\|}_{2}^{2}-{\left\|G_{T_{n1}}-G_{T_{n2}}\right\|}_{2}^{2}+\alpha_{2}\right), \end{array}$$

where *V*_*q*_ is a query instance from the image modality, *T*_*p*_, *T*_*n*1_, and *T*_*n*2_ are three retrieval instances from the text modality, and *V*_*q*_ and *T*_*p*_ are semantic similar. While *V*_*q*_ and *T*_*n*1_, *V*_*q*_ and *T*_*n*2_, and *T*_*n*1_ and *T*_*n*2_ are three pairwise instances, and the two instances in each pairwise have distinct semantics. Equation (2) denotes that the distance of hash representations of similar cross-modal pairwise instances should be smaller than that of dissimilar pairwise instances (both from intermodalities and from intramodalities) with a positive margin (*α*_1_ or *α*_2_). This can ensure that similar cross-modal instances have similar hash representations while dissimilar instances have distinct hash representations. By this quadruplet loss, the cross-modal semantic relevance can be preserved during the hash representation learning stage.

Similarly, given a quadruplet (*T*_*q*_, *V*_*p*_, *V*_*n*1_, *V*_*n*2_), we can have the following cross-modal quadruplet loss:

(3) $$\begin{array}{c} J_{\text{quadruplet}}^{T⟶I}\left(G_{T_{q}},F_{V_{p}},F_{V_{n1}},F_{V_{n2}}\right)=\sum_{T_{q},V_{p},V_{n1}}\max\left(0,{\left\|G_{T_{q}}-F_{V_{p}}\right\|}_{2}^{2}-{\left\|G_{T_{q}}-F_{V_{n1}}\right\|}_{2}^{2}+\alpha_{3}\right)\\ +\sum_{T_{q},V_{p},V_{n1},V_{n2}}\max\left(0,{\left\|G_{T_{q}}-F_{V_{p}}\right\|}_{2}^{2}-{\left\|F_{V_{n1}}-F_{V_{n2}}\right\|}_{2}^{2}+\alpha_{4}\right), \end{array}$$

where *T*_*q*_ is a query instance from the text modality, *V*_*p*_, *V*_*n*1_, and *V*_*n*2_ are three retrieval instances from the image modality, *G*_*T*_*q*__, *F*_*V*_*p*__, *F*_*V*_*n*1__, and *F*_*V*_*n*2__ are hash representations for instances *T*_*q*_, *V*_*p*_, *V*_*n*1_, and *V*_*n*2_, respectively, and *α*_3_ and *α*_4_ are two positive margins. Equation (3) is distinct to equation (2) as the modality of query instance and the modality of retrieval instances are inverse.

### 2.3. Hash Representation Learning and Hash Code Learning

For each quadruplet from training set, it is easy to learn their hash representations and fully protect the semantic similarity with the above quadruplet-based cross-modal semantic relevance preserving module, so we have the following hash representation learning loss:

(4) $$\begin{array}{c} J_{\text{representation}}=\frac{1}{n_{I⟶T}}J_{\text{quadruplet}}^{I⟶T}\left(F_{V_{q}},G_{T_{p}},G_{T_{n1}},G_{T_{n2}}\right)+\frac{\beta }{n_{T⟶I}}J_{\text{quadruplet}}^{T⟶I}\left(G_{T_{q}},F_{V_{p}},F_{V_{n1}},F_{V_{n2}}\right), \end{array}$$

where *n*_*I*⟶*T*_ is the number of quadruplets for utilizing image to retrieve text, *n*_*T*⟶*I*_ is the number of quadruplets for utilizing text to retrieve images, and *β* is a hyperparameter to balance the two parts.

Additionally, to learn high-quality hash codes, we generate hash codes from the learned hash representations with the sign function in equation (1), and the final hash codes matrix for all training image-text pairs are generated as follows:

(5) $$\begin{array}{c} B=\text{sign}\left(\frac{F+G}{2}\right). \end{array}$$

As *F* and *G* are real-valued features, to decrease the information loss from *F* and *G* to *B* in equation (5), it is necessary to force *F* and *G* to be as close as possible to *B*; thus, we introduce the following quantization loss:

(6) $$\begin{array}{c} J_{\text{quantization}}=\frac{{\left\|B-F\right\|}_{2}^{2}+{\left\|B-G\right\|}_{2}^{2}}{2nk}. \end{array}$$

Integrating the hash representation loss and the quantization loss together, the whole loss function is as follows:

(7) $$\begin{array}{c} J=J_{\text{representation}}+\gamma J_{\text{quantization}}, \end{array}$$

where *γ* is a hyperparameter to balance the hash representation loss and the quantization loss.

### 2.4. Feature Extraction Networks

In QDCMH, feature extraction includes two deep neural networks: a classical convolutional neural network is used to extract the features of images and a multiscale fusion model is utilized to learn features from texts. Specifically, for image modality, we deploy AlexNet [11] pretrained on the ImageNet [19] dataset. We then fine-tune the last layer using a new fully connected hash layer which consists of *k* hidden nodes. Therefore, the learned deep features have been embedded into a *k*-dimensional Hamming space. For text modality, the TxtNet in SSAH [15] is used, which comprises a three-layer feedforward neural network and a multiscale (MS) fusion model (Input⟶MS⟶4096⟶512⟶*k*).

## 3. Learning Algorithm of QDCMH

For QDCMH, we utilize alternating strategy to learn parameters *θ*_*V*_ of deep neural networks for image modality and parameters *θ*_*T*_ of deep neural networks for text modality and hash codes matrix *B* for all training image-text pairs. When we learn one of *θ*_*V*_, *θ*_*T*_, and *B*, we keep the other two fixed. The specific algorithm for QDCMH is depicted in Algorithm 1.

### 3.1. Update *θ*_*V*_ with *θ*_*T*_ and *B* Fixed

When *θ*_*T*_ and *B* are maintained fixed, we utilize stochastic gradient descent and backpropagation to optimize the deep neural network parameters *θ*_*V*_.

### 3.2. Update *θ*_*T*_ with *θ*_*V*_ and *B* Fixed

When we fix the values of *θ*_*V*_ and *B*, we use stochastic gradient descent and backpropagation to learn the deep neural network parameters *θ*_*T*_.

### 3.3. Update *B* with *θ*_*T*_ and *θ*_*V*_ Fixed

When the deep neural networks' parameters *θ*_*T*_ and *θ*_*V*_ are kept unchanged, the hash codes matrix *B* can be optimized with equation (5).

## 4. Experiments

### 4.1. Datasets

To investigate the performance of QDCMH, we conduct experiments on two benchmark cross-modal retrieval datasets: MIRFLICKR-25K [20] and Microsoft COCO2014 [21], and the brief descriptions of the datasets are listed in Table 1.

### 4.2. Evaluation Metrics

In our experiments, we utilize mean average precision (MAP), top *N*-precision curves (top *N* Curves), and precision-recall curves (PR Curves) as evaluation metrics; for the detailed description of these evaluation metrics, refer to [22, 23].

### 4.3. Baselines and Implementation Details

We compare our proposed QDCMH method with eight state-of-the-art cross-modal hashing methods, including four handcrafted ones, i.e., cross-modal similarity sensitive hashing (CMSSH) method [7], semantics-preserving hashing (SePH) [9] method, semantic correlation maximization (SCM) method [8], and generalized semantic preserving hashing (GSPH) method [10] and four deep feature-based ones, i.e., deep cross-modal hashing (DCMH) method [13], pairwise relationship guided deep hashing (PRDH) method [14], self-supervised adversarial hashing (SSAH) method [15], and triplet-based deep hashing (TDH) method [16]. Most baseline methods are carefully implemented based on the codes provided by the authors. A few baseline methods are implemented by us following the suggestions and descriptions of the original papers.

All the experiments are executed by using the open source deep learning framework pytorch and running on an NVIDIA GTX Titan XP GPU server. In our experiments, we set *n*_*I*⟶*T*_=*n*_*T*⟶*I*_=10000, max_epoch=500, and *λ*=10^−5^ and the learning rate is initialized to 10^−1.5^ and gradually decreased to 10^−6^ in 500 epochs. For those handcrafted feature-based baselines, each image in the two datasets is represented by a bag of words (BoW) histogram or feature vector having 512 dimensions. For the whole experiment, we use *I*⟶*T* to denote using a querying image while returning text and *T*⟶*I* to denote using a querying text while returning an image.

### 4.4. Performance Evaluation and Discussion

**Figure 3:**
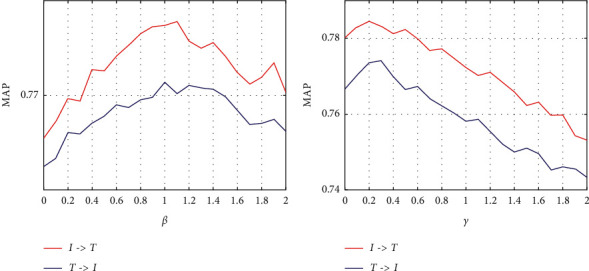
A sensitivity analysis of the hyperparameters. (a) Hyperparameter *β* on MIRFLICKR-25K dataset. (b) Hyperparameter *γ* on MIRFLICKR-25K dataset.

Firstly, we investigate the performance of QDCMH with different hyperparameters *β* and *γ*. To this goal, we experiment on MIRFLICKR-25K with the hash code length *k*=64 and record the corresponding MAPs under different values of *β* and *γ*, as shown in Figure 3. We find that high performance can be acquired when *β*=1 and *γ*=0.2.

Secondly, to validate the performance of QDCMH, we perform the experiment to compare QDCMH with baseline methods in terms of MAP on datasets MIRFLICKR-25K and MS-COCO2014.  Table 2 presents the MAPs of each method for different hash code lengths, i.e., 16, 32, and 64. DSePH represents the SePH method whose features of the original images are extracted by CNN–F. From Table 2, we can see that the following. (1) The MAPs of our proposed QDCMH are higher than the MAPs of most baseline methods in most cases, which demonstrates the superiority of QDCMH. We can also observe that SSAH outperforms than our proposed QDCMH in most cases, which is partly because SSAH takes self-supervised learning and generative adversarial networks into account during hash representation learning procedure. (2) The MAPs of QDCMH is always higher than the MAPs of TDH, which shows that quadruplet loss can better preserve semantic relevance than triplet loss in cross-modal hashing retrieval. (3) The MAPs of DSePH is always higher than the MAPs of SePH, which demonstrates that deep neural networks have powerful features learning capacity. (4) Our proposed QDCMH can achieve better performance on MS-COCO 2014 dataset than on MIRFlickr-25K dataset, which is partly because the instances in MS-COCO 2014 dataset belong to 80 categories while the instances in MIRFlickr-25K dataset belong to 24 categories, and this makes the quadruplets generated from the MS-COCO 2014 dataset have better generalization ability than the quadruplets generated from the MIRFlickr-25K dataset.

**Figure 4:**
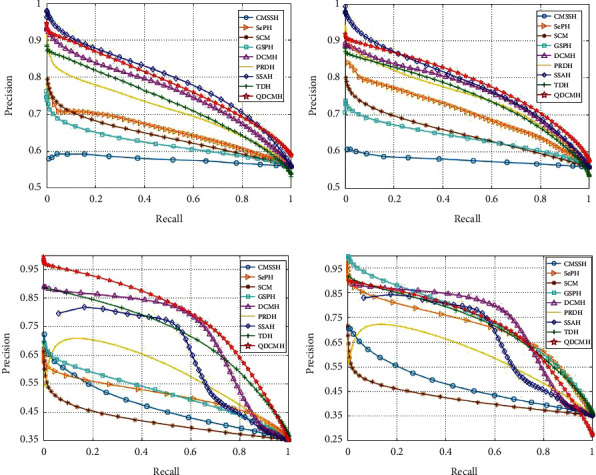
Precision-recall curves on datasets MIRFLICKR-25K and Microsoft COCO2014.

**Figure 5:**
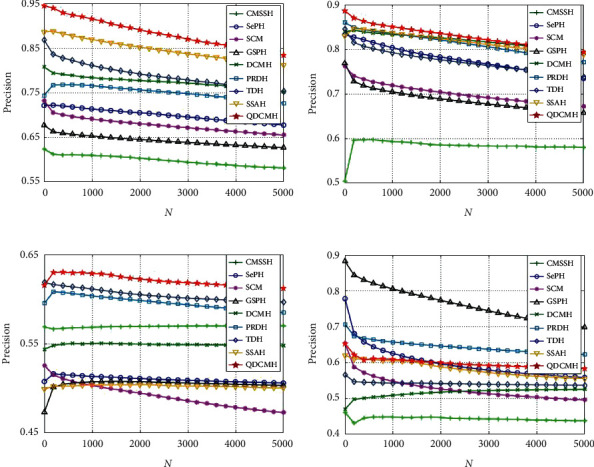
Top *N*-precision curves on datasets MIRFLICKR-25K and Microsoft COCO2014.

Thirdly, to further investigate the performance of QDCMH, we plot the precision-recall curves and top *N*-precision curves of QDCMH and baseline methods with hash code lengths 64 on datasets MIRFLICKR-25K, Microsoft COCO2014, respectively, as presented in Figures 4 and 5. From this figure, we can see that the precision-recall curves and top *N*-precision curves are nearly consistent with the MAPs in Table 2.

## 5. Conclusions

In this paper, we introduce a quadruplet loss into deep cross-modal hashing to fully preserve semantic relevance of original cross-modal quadruple instances and propose a quadruplet based deep cross-modal hashing method (QDCMH). QDCMH integrates quadruplet-based cross-modal semantic relevance preserving module, hash representation learning, and hash code generation into an end-to-end framework. Experiments on two benchmark cross-modal retrieval datasets demonstrate the efficiency of our proposed QDCMH.

## Figures and Tables

**Algorithm 1:**
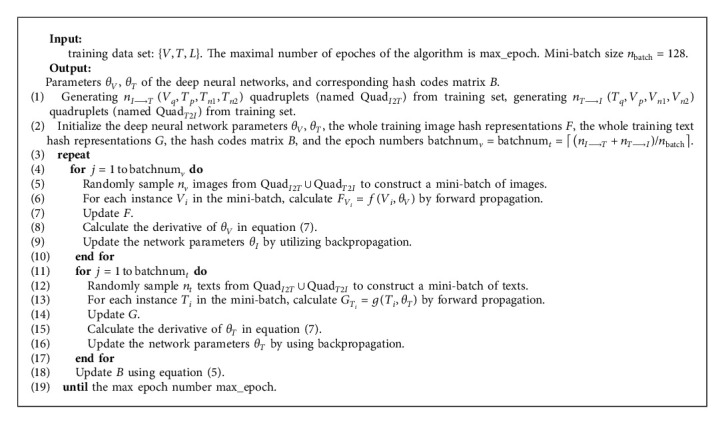
QDCMH: quadruplet-based deep cross-modal hashing.

**Table 1:** Brief description of the experimental datasets.

Dataset	Used	Train	Query	Retrieve	Tag dimension	Labels
MIRFLICKR-25K	20,015	10,000	2,000	18,015	1,386	24
MS-COCO2014	122,218	10,000	5,000	117,218	2,026	80

**Table 2:** Comparison to baselines in terms of MAP on two datasets: MIRFLICKR-25K, and Microsoft COCO2014, respectively. The best accuracy is shown in boldface.

Task	Methods		MIRFlickr-25K			MS-COCO		
			16bits	32bits	64bits	16bits	32bits	64bits
I⟶T	Handcrafted methods	CMSSH [7]	0.5600	0.5709	0.5836	0.5439	0.5450	0.5410
		SePH [9]	0.6740	0.6813	0.6803	0.4295	0.4353	0.4726
		SCM [8]	0.6354	0.6407	0.6556	0.4252	0.4344	0.4574
		GSPH [10]	0.6068	0.6191	0.6230	0.4427	0.4733	0.4840
	Deep methods	DCMH [13]	0.7316	0.7343	0.7446	0.5228	0.5438	0.5419
		PRDH [14]	0.6952	0.7072	0.7108	0.5238	**0.5521**	**0.5572**
		SSAH [15]	**0.7745**	**0.7882**	**0.7990**	0.5127	0.5256	0.5067
		TDH [16]	0.7423	0.7478	0.7512	0.5164	0.5222	0.5276
		DSePH [9]	0.7128	0.7285	0.7422	0.4621	0.4958	0.5112
		QDCMH	0.7635	0.7688	0.7713	**0.5286**	0.5313	0.5371

T⟶I	Handcrafted methods	CMSSH [7]	0.5726	0.5776	0.5753	0.3793	0.3876	0.3899
		SePH [9]	0.7139	0.7258	0.7294	0.4348	0.4606	0.5195
		SCM [8]	0.6340	0.6458	0.6541	0.4118	0.4183	0.4345
		GSPH [10]	0.6282	0.6458	0.6503	0.5435	0.6039	0.6461
	Deep methods	DCMH [13]	0.7607	0.7737	0.7805	0.4883	0.4942	0.5145
		PRDH [14]	0.7626	0.7718	0.7755	0.5122	0.5190	0.5404
		SSAH [15]	**0.7860**	**0.7974**	**0.7910**	0.4832	0.4831	0.4922
		TDH [16]	0.7516	0.7577	0.7634	0.5198	0.5332	0.5399
		DSePH [9]	0.7422	0.7578	0.7760	0.4616	0.4882	0.5305
		QDCMH	0.7762	0.7725	0.7859	**0.5245**	**0.5398**	**0.5487**

## Data Availability

The experimental datasets and the related settings can be found in https://github.com/SWU-CS-MediaLab/MLSPH. The experimental codes used to support the findings of this study will been deposited in the github repository after the publication of this paper or can be provided by xitaozou@sanxiau.edu.cn.
